# Supplementary figures and images for: Creation of Novel Protein Variants with CRISPR/Cas9-Mediated Mutagenesis: Turning a Screening By-Product into a Discovery Tool
Source: PLoS One. 2017 Jan 24;12(1):e0170445. doi: 10.1371/journal.pone.0170445 (PMC5261743; doi:10.1371/journal.pone.0170445)

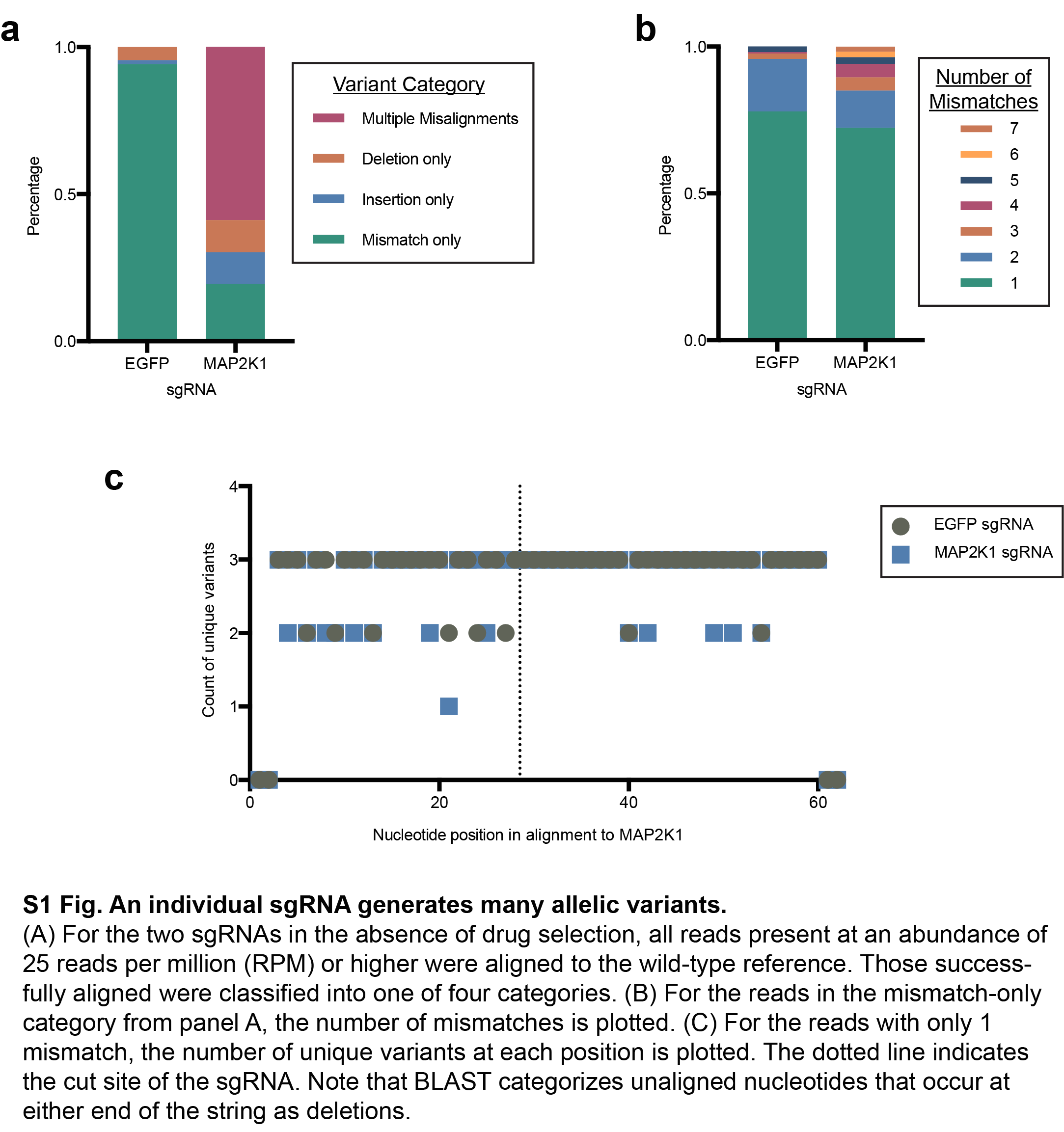

Supplement: S1 Fig — (A) For the two sgRNAs in the absence of drug selection, all reads present at an abundance of 25 reads per million (RPM) or higher were aligned to the wild-type reference. Those successfully aligned were classified into one of four categories. (B) For the reads in the mismatch-only category from panel A, the number of mismatches is plotted. (C) For the reads with only 1 mismatch, the number of unique variants at each position is plotted. The dotted line indicates the cut site of the sgRNA. Note that BLAST categorizes unaligned nucleotides that occur at either end of the string as deletions. (TIF) [file pone.0170445.s001.tif]

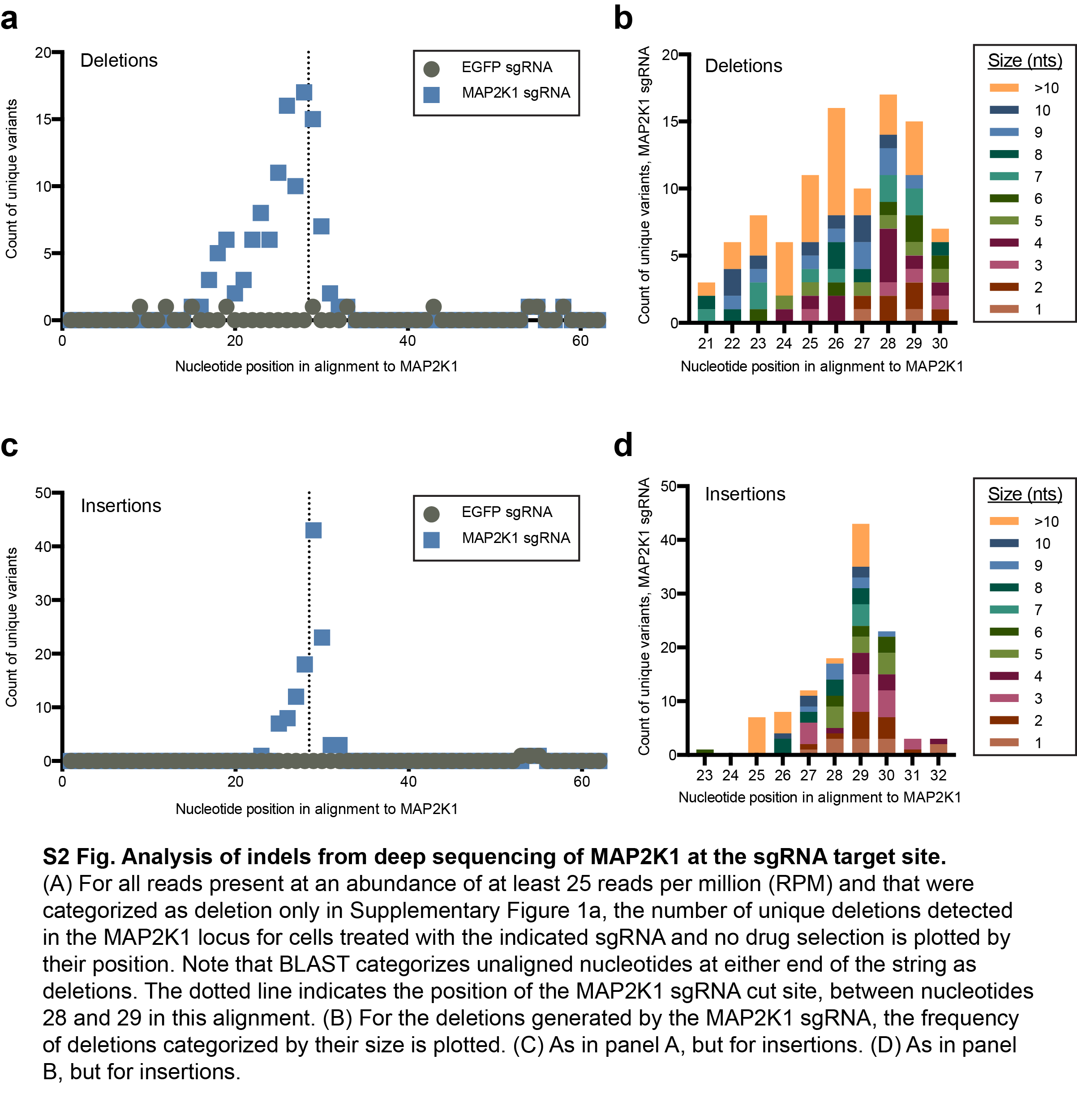

Supplement: S2 Fig — (A) For all reads present at an abundance of at least 25 reads per million (RPM) and that were categorized as deletion only in S1A Fig, the number of unique deletions detected in the MAP2K1 locus for cells treated with the indicated sgRNA and no drug selection is plotted by their position. Note that BLAST categorizes unaligned nucleotides at either end of the string as deletions. The dotted line indicates the position of the MAP2K1 sgRNA cut site, between nucleotides 28 and 29 in this alignment. (B) For the deletions generated by the MAP2K1 sgRNA, the frequency of deletions categorized by their size is plotted. (C) As in panel A, but for insertions. (D) As in panel B, but for insertions. (TIF) [file pone.0170445.s002.tif]
